# Total hip arthroplasty for a woman with hemophilia A -case report-

**DOI:** 10.1016/j.amsu.2019.05.003

**Published:** 2019-05-24

**Authors:** Akio Kanda, Kazuo Kaneko, Osamu Obayashi, Atsuhiko Mogami, Itaru Morohashi

**Affiliations:** a Department of Orthopaedic Surgery, Juntendo Shizuoka Hospital, Izunagaoka 1129, Izunokuni-country, 410-2295, Shizuoka, Japan; b Department of Orthopaedic Surgery, Juntendo University, Hongou3-1-3, Bunkyou Ward, 113-8431, Tokyo, Japan; c Department of Orthopaedic Surgery, Juntendo Shizuoka Hospital, Nagaoka 1129, Izunokuni-country, 410-2295, Shizuoka, Japan

**Keywords:** Clotting factor VIII, Female hemophilia A carrier, Total hip arthroplasty

## Abstract

Hemophilia A is a congenital bleeding disorder caused by an X-linked hereditary pattern. Female hemophilia A carriers are usually asymptomatic, although some have far lower levels of clotting factor because more X chromosomes with the normal gene are switched off, a phenomenon referred to as "lyonization.” During a medical checkup at our hospital, a 56-year-old Japanese woman with coxalgia was also diagnosed as an obligate hemophilia A carrier based on World Federation of Hemophilia criteria. She underwent total hip arthroplasty using blood product coagulation factor VIII to address her hemophilia. Immediate female relatives (mother, sisters, daughters) of a person with hemophilia should have their clotting factor levels checked, especially prior to any invasive intervention or childbirth, or if any symptoms occur.

## Highlights

• We report a case of total hip arthroplasty for a woman with hemophilia A.
• Some female hemophilia A carriers have far lower levels of clotting factor.
• Immediate female relatives of a person with hemophilia should have their clotting factor level checked.

## 1 Introduction

Hemophilia A is a congenital bleeding disorder caused by an X-linked hereditary pattern and a deficiency in clotting factor VIII [1]. Approximately 400,000 people worldwide have hemophilia [2]. X-linked recessive congenital disorders tend to occur primarily in male infants, although female newborns are occasional carriers of the hemophilia A gene. They are usually asymptomatic, however, because the presence of a single copy of normal factor VIII suffices to ensure the production of a sufficient concentration of factor VIII for hemostasis [3]. On average, hemophilia carriers have about 50% of the normal amount of clotting factor because the hemophilia A gene is “turned off” (inactivated) in about half of their cells. Some carriers have far lower levels of clotting factor because more of the X chromosomes with the normal gene are switched off—i.e., "lyonization" has taken place [4]. A definitive diagnosis depends on a factor assay to reveal a factor VIII deficiency. A woman who has <40% of the normal level of clotting factor is no different from a man with the same factor levels—i.e., she also manifests hemophilia [4]. Persons with mild hemophilia, however, are rarely troubled with bleeding during everyday life. Hence, many persons have undiagnosed hemophilia and, with their lack of this clotting factor activity, undergo surgery that causes unexpected massive bleeding. It should therefore be mandatory to measure a patient's factor VIII activity and, if low, to supplement it when faced with surgery, delivery, or other invasive activity.

This case study was performed and is being reported in line with the SCARE criteria [5].

## 2 Presentation of the case

**Fig. 1:**
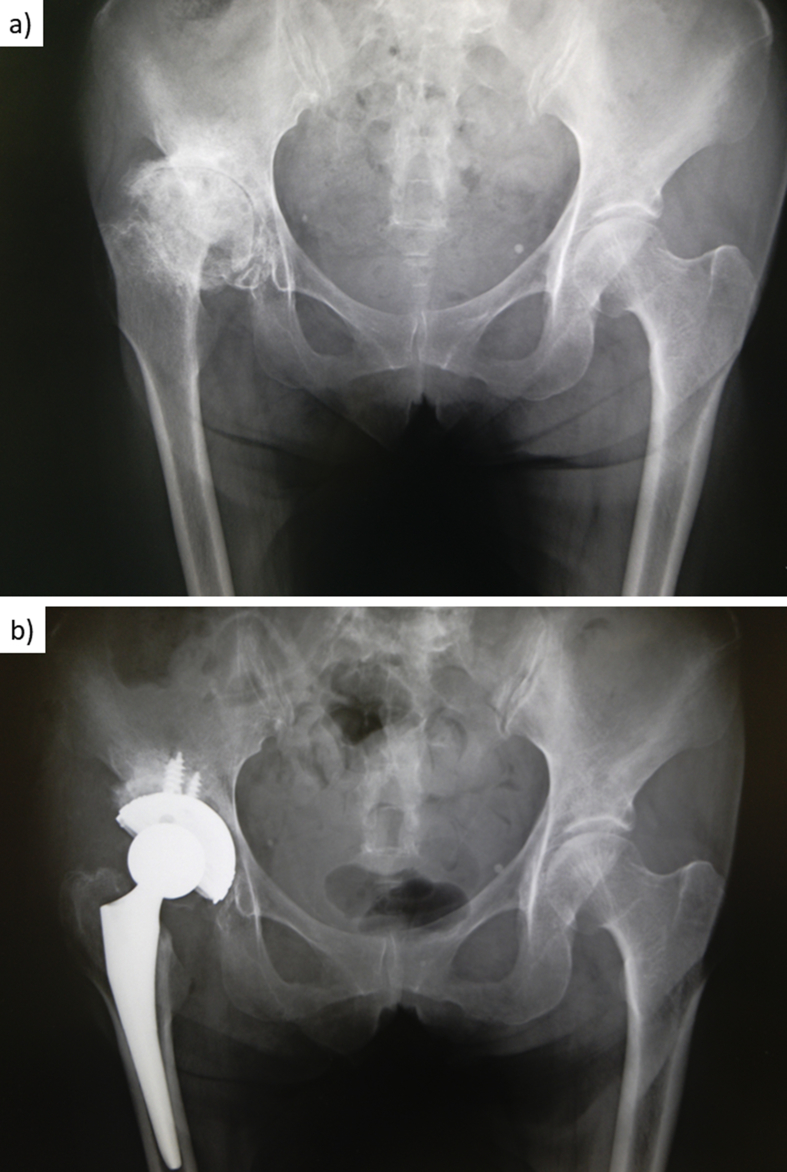
(**a**) Preoperative plain radiograph. The patient had developed coxarthrosis and severe pain of the right hip joint. Radiographs show stage 4 arthritis based on the Japanese Orthopaedic Association hip score. (**b**) Postoperative plain radiograph, which confirmed the implant's correct setting.

A 56-year-old Japanese woman who had developed coxarthrosis and severe pain of the right hip joint (Fig. 1a) underwent a medical checkup at our hospital. She had not experienced hemarthrosis and had not been diagnosed with hemophilic arthropathy. She was found to have secondary coxarthrosis with dysplasia of the acetabula. We planned total hip arthroplasty (THA). Her history was disturbing, however, as neonatally she had experienced congenital hip dislocation, for which she underwent surgery that required massive blood transfusion. Then, at 12 years of age, she was operated on for appendicitis and again required transfusion for massive bleeding. Soon afterward, her newborn nephew was diagnosed with hemophilia by a pediatrician who had detected a family history of hemophilia through a younger sister. Our patient's younger and older brothers had died from massive bleeding but had not been diagnosed with hemophilia A.

A pediatrician then diagnosed our patient as having hemophilia A (i.e., coagulation factor VIII deficiency). She delivered a boy by cesarean section without undue bleeding because she was given clotting factor VIII supplements. The newborn boy was diagnosed with severe hemophilia A. Because the patient's son and her nephew were diagnosed with severe hemophilia A, our patient was diagnosed as an obligate carrier based on the classification of the World Federation of Hemophilia [1]. Her clotting factor VIII comprised 14.7% of the normal amount of clotting factor VIII, and her hemophilia A was deemed mild.

Her preoperative blood examination did not show a prolongation activated partial thromboplastin time, which is pathognomonic for hemophilia A. She had no medical history of drug taking, psychiatric problems, or familial abnormalities. Our hospital hematologist advised the use of the blood product coagulation factor VIII and fresh frozen plasma (FFP). Preoperatively, the patient had about 14.7% of the normal amount of coagulation factor VIII. We also checked for the absence of an inhibitor.

Each unit of factor VIII per kilogram body weight infused intravenously elevates the plasma factor VIII level by approximately 2 IU/dl. The half-life of factor VIII is approximately 8–12 h. The dose is calculated according to the following equation [1].

Patient's weight (kg) × desired rise in factor level (IU/dl) × 0.5.

**Fig. 2:**
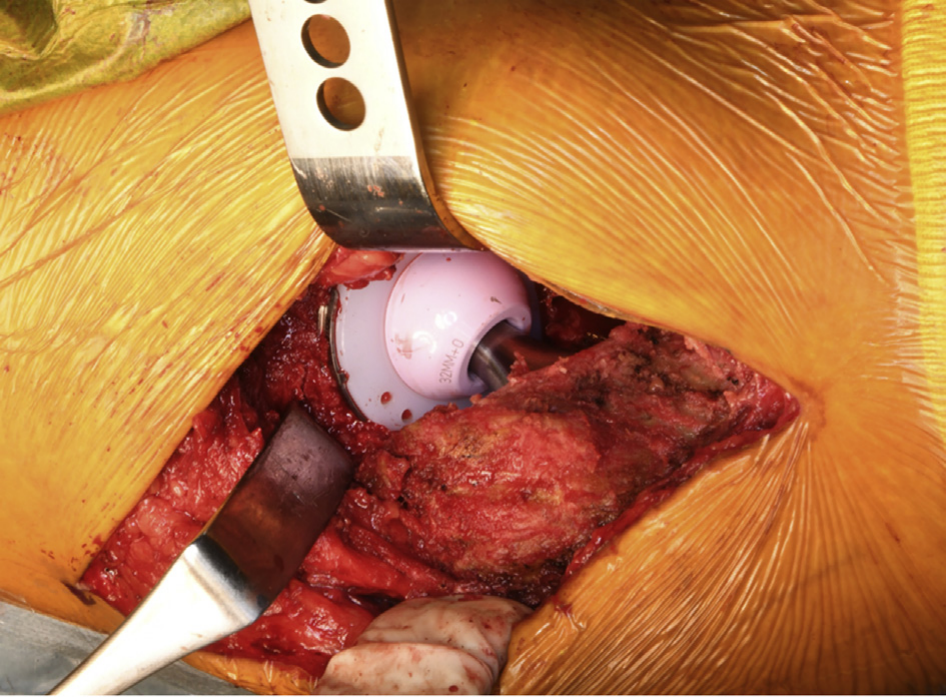
Intraoperative photograph. We performed total hip arthroplasty using a direct lateral approach. No abnormal bleeding occurred during the operation.

This patient weighed 50 kg, and the dosage aim for preoperative factor VIII for major surgery was 80%–100% [1]. Therefore, we gave [50 kg × (100–14.2) × 0.5] units—i.e., about 2000 units—of blood product coagulation factor VIII to the patient 2 h before the surgery. During the operation, if massive bleeding occurred, we planned to give immediate additional FFP and blood product coagulation factor VIII. We performed THA after explaining the use of clotting factors for bleeding control to the patient. Perioperative bleeding amounted to 120 g. The orthopedic surgeon who performed the THA had 18 years of experience with this operation. There was no abnormal bleeding intraoperatively (Fig. 2). Correct setting of the implant was confirmed by postoperative radiography (Fig. 1b).

We administered an additional 2000 units of blood product coagulation factor VIII postoperatively because the half-life of factor VIII is approximately 8–12 h. At 16 h postoperatively, there was 155 g of drained blood. Following such major surgery, during postoperative days (PODs) 1–3, the aim was to decrease the factor VIII dose 60%–80%; on PODs 4–14, 40%–60%; and on PODs 7–14, 30–50%. Thus, we gave 1000 units of blood product coagulation factor VIII twice a day to the patient on PODs 1–8 and 1500 units of blood product coagulation factor VIII once a day on PODs 9–12. We checked the coagulation factor VIII levels several times. There was at least a 1-week period between the time blood was obtained for the test and when we received the results, so we checked hemorrhagic status via an outflow of blood from a drain, femoral swelling, and anemia progression via blood testing. Postoperative bleeding diminished without a problem (Table 1). However, the targeted factor VIII dose level was not reached during the operation or on the day after the surgery. We did not set a limited follow-up duration after the THA. At 6 months postoperatively, however, she had not experienced any hemorrhaging.

**Table 1:**
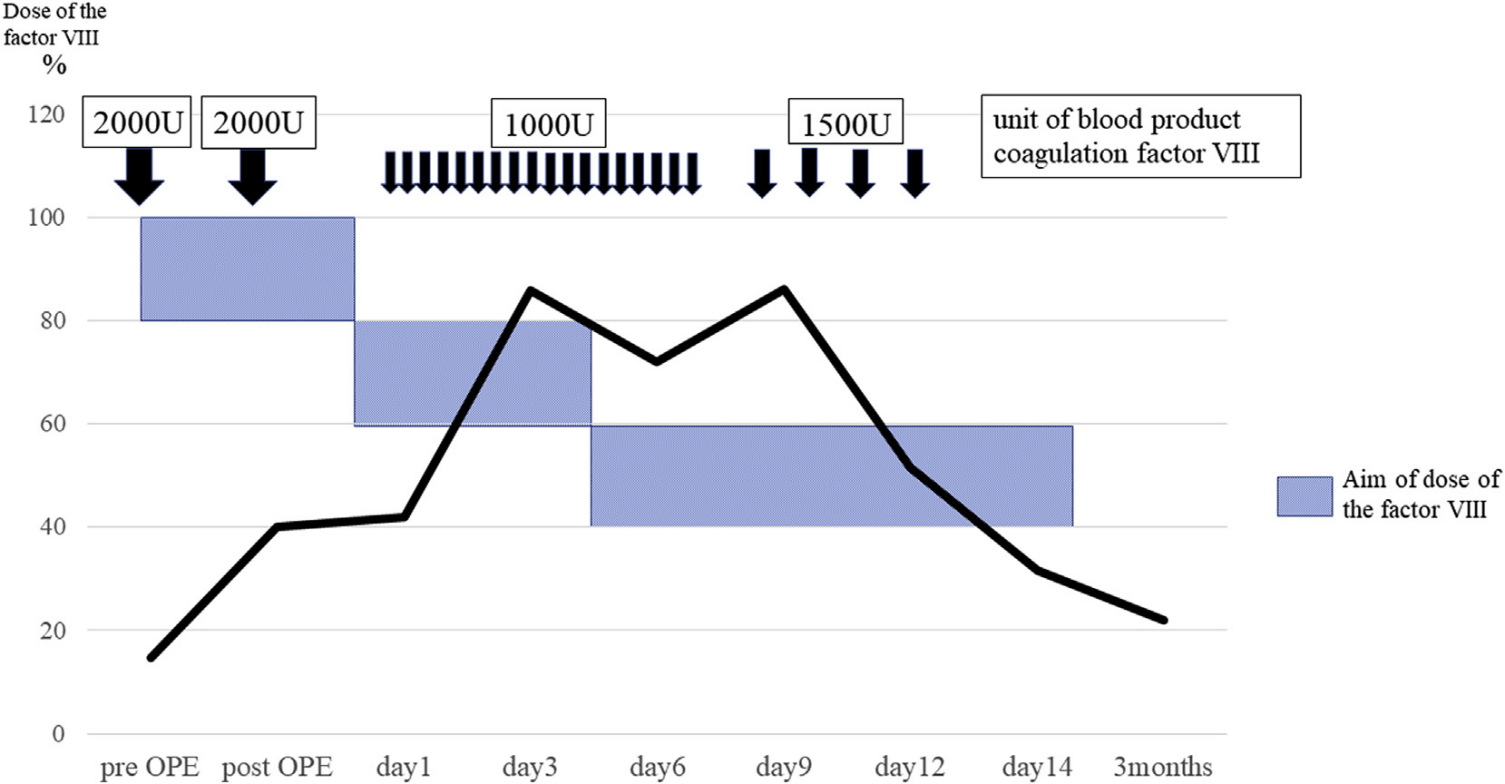
Factor VIII doses and amounts of the blood product coagulation factor VIII required.

## 3 Discussion

Female hemophilia A carriers are usually asymptomatic because the presence of a single copy of normal factor VIII ensures the production of sufficient amounts of factor VIII for hemostasis [3]. Some carriers have far lower levels of clotting factor because many X chromosomes with the normal gene are switched off (i.e., lyonized) [4]. A definitive diagnosis depends on a factor assay to identify factor VIII deficiency. Female carriers can be diagnosed with hemophilia A. Additionally, moderate or mild hemophilia does not produce hemorrhagic symptoms during normal life events. However, when clotting factor activity is decreased during surgery, massive bleeding may occur.

Our patient had not experienced hemarthrosis, and she had secondary coxarthrosis due to dysplasia of the acetabula, not hemophilic arthropathy. During her previous surgeries, however, she had required massive blood transfusion on a scale not usually required for such bleeding. Fortunately, around this time, a pediatrician evaluated her family history, which revealed massive bleeding in other family members, suggesting hemophilia. With this knowledge we were able to perform THA without massive bleeding. Because her factor VIII activity was only 40.2% postoperatively, we gave her an additional 2000 units of blood product coagulation factor VIII. During the surgery, performed the next day, her coagulation factor VIII activity increased to 42.0% before we gave an additional 1000 units of blood product coagulation factor VIII. Afterward, her factor VIII activity stabilized at 80%, from 70%. No perioperative massive bleeding occurred. However, because her factor VIII activity immediately after the operation was low, we increased the dosage of blood product coagulation factor VIII.

## 4 Conclusion

The patient's blood examination showed no abnormalities, such as a prolonged activated partial thromboplastin time (which is pathognomonic for hemophilia A). Hence, it was vital to consider hemophilia A, and we therefore measured her factor VIII activity. The results indicated a need to supplement clotting factor VIII. Thus, the immediate female relatives (mother, sisters, daughters) of a person with hemophilia should have their clotting factor level checked, especially prior to any invasive intervention or childbirth, or if any symptoms occur [1]. Ignoring such advice could result in massive, death-threatening bleeding during total hip replacement.

## Consent of patient

Written informed consent was obtained from the patient for publication of this case report and any accompanying images.

## Ethical approval

This case report is written based on institutional ethical committee.

## Sources of funding

No funds were received in support of this study. No benefits in any form have been or will be received from a commercial party related directly or indirectly to the subject of this manuscript.

## Author contribution

AK conceived the study, participated in its design and coordination, and drafted the manuscript. KK helped to draft the manuscript. OO helped to draft the manuscript. AM helped to draft the manuscript. IM helped to draft the manuscript. All authors read and approved the final manuscript.

## Conflicts of interest

The authors stated that they had no interests which might be perceived as posing a conflict or bias.

## Trial registry number

None.

## Guarantor

Akio Kanda.

## Conflicts of interest

No funds were received in support of this study. No benefits in any form have been or will be received from a commercial party related directly or indirectly to the subject of this manuscript.

## Author contribution

Please specify the contribution of each author to the paper, e.g. study design, data collections, data analysis, writing. Others, who have contributed in other ways should be listed as contributors. AK conceived the study, participated in its design and coordination, and drafted the manuscript. KK helped to draft the manuscript. OO helped to draft the manuscript. AM helped to draft the manuscript. IM helped to draft the manuscript. All authors read and approved the final manuscript.

## Provenance and peer review

Not commissioned, externally peer reviewed.

## Appendix A Supplementary data

Supplementry Supplementry
